# Improving measles incidence inference using age-structured serological data

**DOI:** 10.1017/S0950268818002054

**Published:** 2018-08-06

**Authors:** Joaquin M. Prada, C. Jessica E. Metcalf, Matthew J. Ferrari

**Affiliations:** 1 Faculty of Health and Medical Sciences, University of Surrey, Guildford, UK; 2 Department of Ecology and Evolutionary Biology, Princeton University, Princeton, New Jersey, USA; 3 Office of Population Research, WWS, Princeton University, Princeton, New Jersey, USA; 4 Center for Infectious Disease Dynamics, Pennsylvania State University, State College, Pennsylvania, USA

**Keywords:** Infectious disease epidemiology, measles (rubeola), public health, rubella

## Abstract

Measles is a target for elimination in all six WHO regions by 2020, and over the last decade, there has been considerable progress towards this goal. Surveillance is recognised as a cornerstone of elimination programmes, allowing early identification of outbreaks, thus enabling control and preventing re-emergence. Fever–rash surveillance is increasingly available across WHO regions, and this symptom-based reporting is broadly used for measles surveillance. However, as measles control increases, symptom-based cases are increasingly likely to reflect infection with other diseases with similar symptoms such as rubella, which affects the same populations, and can have a similar seasonality. The WHO recommends that cases from suspected measles outbreaks be laboratory-confirmed, to identify ‘true’ cases, corresponding to measles IgM titres exceeding a threshold indicative of infection. Although serological testing for IgM has been integrated into the fever–rash surveillance systems in many countries, the logistics of sending in every suspected case are often beyond the health system's capacity. We show how age data from serologically confirmed cases can be leveraged to infer the status of non-tested samples, thus strengthening the information we can extract from symptom-based surveillance. Applying an age-specific confirmation model to data from three countries with divergent epidemiology across Africa, we identify the proportion of cases that need to be serologically tested to achieve target levels of accuracy in estimated infected numbers and discuss how this varies depending on the epidemiological context. Our analysis provides an approach to refining estimates of incidence leveraging all available data, which has the potential to improve allocation of resources, and thus contribute to rapid and efficient control of outbreaks.

## Introduction

In sub-Saharan African countries, measles remains one of the leading causes of child morbidity and mortality [1]. Fever–rash case-based surveillance for measles is active in 44 countries out of the 47 WHO member states in the WHO African Region, and in each country, laboratory capacity adequate to run required serological tests also exists [2]. Fever–rash symptoms are caused by a wide array of infections in infants and adolescents (e.g. rubella). Clinically compatible measles cases, defined as cases presenting with fever and rash plus one of either cough, coryza or conjunctivitis, are highly sensitive, and thus useful for detecting outbreaks and triggering reactive interventions. However, in the absence of serological confirmation, this definition may not reflect ‘true’ measles cases, and thus lead to spurious inference about measles dynamics and the impact of control efforts. Moreover, expanding laboratory confirmation through serology is often logistically challenging in resource-poor settings, given the costs of transportation, laboratory equipment and personnel. Overall, surveillance based on clinical fever–rash symptoms alone is fast and inexpensive but can lead to false positives; surveillance based on laboratory confirmation is highly specific, but expensive and logistically challenging. Here, we propose that an efficient pairing of clinical and laboratory-confirmed surveillance may greatly strengthen inference of measles dynamics at the population scale.

The age distribution of measles cases scales with its prevalence. When measles is common, cases are concentrated in younger individuals. As measles becomes less common (e.g. due to vaccination), the mean and variance of the age distribution of cases in endemic countries increase [3, 4]. Many other causes of fever–rash symptoms in children also become less prevalent with age and vary in prevalence from place to place [5, 6]. One example is rubella, which is also a likely cause of fever–rash in these countries. Together, these features mean that the likelihood that an individual presenting with fever–rash symptoms is infected with measles will depend on age and the epidemiological context. The proportion of laboratory-tested cases of fever–rash that are confirmed as measles-positive using measles-specific IgM titres can be used to infer both the overall likelihood that a suspected measles case is a true measles case and the age-specific variation in this likelihood. As measles vaccination coverage increases, the likelihood of fever–rash symptoms being caused by another disease, such as rubella, also increases. Here, we propose a novel model for combining the age-specific serological confirmation probability, with surveillance data based on clinical presentation alone, in order to infer the distribution of confirmed measles and rubella cases. The power and utility of our method is that it allows serological confirmation applied to only a subset of clinically compatible cases to be used to infer the distribution of an infection (here we focused on measles and rubella, but the principles should hold cross-infections) across a broader population, by leveraging the patterns of age incidence. Moreover, in the datasets we analysed, individuals who tested negative for measles IgM were also tested for rubella, which allows us to also estimate age incidence of this infection. We further note that IgM testing has the dual benefit of improving the specificity of measles surveillance and providing insights into rubella dynamics, which has suffered to date from insufficient confirmation.

**Fig. 1.**
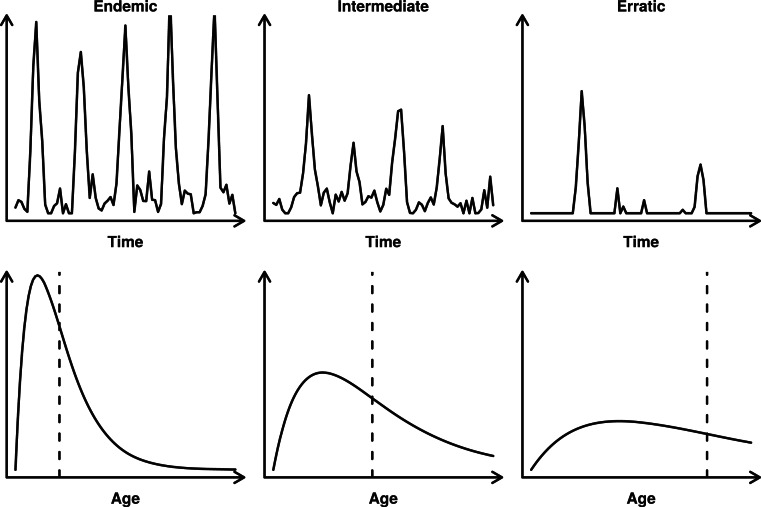
Schematic representation of a measles time series and age incidence from an endemic to erratic setting. In an endemic situation (left), the outbreaks occur periodically and most cases are in young infants. In an erratic setting (right), outbreaks may occur at random; there is no periodicity and the mean age of infection is higher (vertical dashed line).

To illustrate this, we analysed contemporary surveillance datasets across a gradient of measles endemicity from three countries in sub-Saharan Africa: Ethiopia, Kenya and Zimbabwe. WHO/UNICEF Estimates of National Immunization Coverage (WUENIC) [7] indicate that the magnitude of routine measles vaccination coverage varies considerably across these countries. Although all three have experienced increases in vaccination coverage over recent years, the data reflect a gradient of progress, ranging from Ethiopia, with the lowest first dose coverage of 44% in 2003, to Zimbabwe, with coverage of 92% in 2011. Thus, we expect a concomitant gradient in dynamics, from regular endemic transmission in Ethiopia, to episodic outbreaks in Zimbabwe, with Kenya intermediate (Fig. 1). The proportion of suspected measles cases that are sent in for serological confirmation in these datasets varies, ranging from 55% (in Ethiopia) to 95% (in Kenya).

Here, we explore the variability in the country-specific pattern of age-dependent measles and rubella IgM confirmation. We then evaluate how this result can be used to strengthen the inference into temporal patterns of disease incidence by comparing analyses using three different sources of data: (i) all reported measles cases (i.e. all syndromic cases, regardless of whether they were serologically tested or not; henceforth simply referred to as syndromic or symptomatic cases), (ii) serologically confirmed cases only and (iii) the sum of serologically confirmed cases and the fraction of untested syndromic cases inferred to be positive by our model, henceforth referred to as estimated cases.

Below, we first introduce the countries and data sources, then describe our model framework, and characterise the steps taken to estimate true measles/rubella incidence and reconstruct the time series. Finally, we estimate the minimum number of cases that we would need tested to accurately estimate the age distribution.

## Materials and methods

### Data sources

We analysed individual fever–rash case records from Ethiopia, Kenya and Zimbabwe between 2002 and 2014 (see Table 1). Data were provided by the Ministry of Health for Ethiopia; for Zimbabwe it was sourced from Chimhuya *et al*. [8]; Kenya from Wesolowski *et al*. [9]. Each record included the date of onset of symptoms, the age of the individual and the result of a measles and rubella IgM test, if conducted. IgM antibodies specific to measles (or rubella, for rubella infection) reach high titres on the day the rash appears and persist up to 3 months post-infection, and thus provide a strong test of recent infection. Untested individuals were listed as either ‘epidemiologically linked’ or ‘suspected’ cases. The WHO definition of ‘epidemiologically linked’ is broad and includes fever–rash cases in direct contact with a confirmed case or in the same/neighbouring district of at least three confirmed cases (which is considered an outbreak under WHO definition [10]). We treated suspected cases with serological results characterised as ‘unclear’ or otherwise undescribed as being untested. We treat all epidemiologically linked and suspected cases as ‘syndromic’ cases, i.e. that are consistent with measles infection, but not confirmed. For each country, the number of individuals recorded in the dataset, the number that are laboratory-confirmed and the range of the time series are shown in Table 1.

**Table 1.** Summary of datasets used

Country	Population size (millions)	MCV 2014 coverage (%)	*n* entries	*n* tested measles IgM (%)	*n* tested rubella IgM (%)	Range (years)
Ethiopia	Under 100	70	52 282	56	34	2003–2014
Kenya	~45	79	17 625	95	76	2002–2011
Zimbabwe	~14.5	92	3428	87	67	2007–2011

### Model description and validation

#### Age-specific confirmation rate

The probability that a syndromic case is due to measles infection will vary as a function of age and the country context. Measles (and rubella) are immunizing infections; therefore, population susceptibility decreases with age (due to vaccination and natural infection). Moreover, the severity of measles disease declines with age [11], thus older individuals may be less likely to seek care. However, other sources of fever–rash illness, such as rubella, may also be immunizing or have age-dependent incidence, which would increase the rates of fever–rash illness due to non-measles causes in young children [4]. This means that the probability that a syndromic case is due to measles (rather than rubella or other infection) and declines with age cannot be assumed; a flexible framework is thus needed that allows for both decreased and/or increased serological confirmation with age.

We model the probability that a syndromic case is confirmed as IgM-positive for measles as 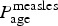, where the confirmation probability in neighbouring age classes is modelled as an autoregressive AR(1) process; i.e. 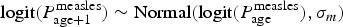 (Supplementary material 1). This generates correlation in the IgM confirmation rate, which can arise either because of correlation in the age-specific likelihood of measles infection, non-measles fever–rash symptoms or health-seeking behaviour, or due to uncertainty in the recording of real age, e.g. an individual who is 58 months of age may have been classified as under 5 or over 5 years. We binned the data into 2-year age classes (total number of classes: *n* = 38). Individuals over 75 years are collapsed to the same (the last) age bin. The bin size was chosen to have multiple bins at low ages (eight bins up to 15 years of age), which are the age groups where the majority of cases were recorded, while at the same time allowing most of the age classes for older individuals to be populated. The number of IgM-positive cases out of all tested syndromic cases was then modelled as binomial, with probability 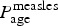. We fit the analogous model, independently, for rubella.

The model was fit in R [12] using the Gibbs sampler package ‘jags’ [13] and ‘runjags’ [14]. Two independent chains were run, with 10 000 samples and a burn in period of 1000. Convergence was verified using the Gelman and Rubin's convergence diagnostic [15] and by visual examination of the chains.

#### Estimation for untested individuals

Some reported cases were not tested for virus-specific IgM, thus we are uncertain whether they are true positives. We estimated the distribution of likely disease positives by resampling from the distribution of suspected cases according to the age-specific probability (above) that a suspected case was IgM-positive. This was done for both measles- and rubella-suspected cases. Given the test is imperfect, we also resampled the IgM-positive/negative cases based on the test's sensitivity/specificity. Sensitivity for the measles IgM test ranges from 87% to 96%; specificity is between 95% and 99%. The sensitivity of the rubella IgM test ranges from 74% to 77%; specificity ranges from 94% to 96%, depending on the commercial assay used [16, 17]. We used the mean values 91% and 97% for the measles test sensitivity and specificity (75% and 95% for rubella) in our model to correct for potential testing errors. Thus, we model the true number of cases in age class *a* as:
1
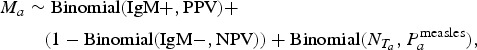
where *M*_*a*_ are the estimated true measles cases in age group *a*; a similar equation can be applied for rubella cases. The first two terms are the resampling based on the positive and negative predictive values, the last term is the sampling of untested individuals in age group *a*, 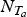. We generated 200 random draws for each age class and present the mean, 2.5th and 97.5th quantiles of those random draws as the point estimate and confidence intervals on the true number of cases. We note that a small number of entries did not have an age recorded. For these cases, we randomly assigned an age based on the empirical age distribution of individuals with known ages.

#### Reconstructing patterns of incidence

We reconstructed the time series for syndromic, serologically confirmed and estimated cases of measles/rubella by pooling the dates of onset to a monthly number of cases. The spectral density was estimated by fitting an AR model, with the order (complexity) chosen by Akaike Information Criterion (AIC), which allows us to recover the seasonality/recurrence of outbreaks if present in the time series, i.e. the main peaks in spectral density; this was done using the ‘spectrum’ function in R [12]. Using the time series based on the estimated number of cases (Equation 1), we calculated the probability of being a measles/rubella case, given that fever–rash symptoms are present, for each month of the time period studied in each country. We can then calculate the average monthly confirmation rate and the variation across the time frame studied for each country.

For each country, we summarise measures of the age distribution (mean, quantiles) and the time series (estimation of the spectral density and average monthly confirmation rate, see below) for (i) all syndromic cases (assumption of no serology being done); for (ii) only those individuals that were IgM-positive; and for (iii) our estimated number of cases (Equation 1), for both measles and rubella. Mean age of infection was defined as 
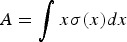
, with *x* being age and *σ*(*x*) the proportion susceptible at age *x* [18], taking 1–(the cumulative proportion of case numbers over age) as a proxy for the proportion susceptible.

#### Model cross-validation

To assess the performance of the model, we evaluated the predictions made for untested individuals using a repeated random sub-sampling validation design. We split the subset of tested individuals, *N*, randomly into two equally sized groups; *T*, tested individuals and *U*, ‘assumed untested’ (so that *N* = *T* + *U*). We initially chose the two groups to be of equal size for the validation, as it is consistent with our Ethiopia dataset, where approximately half of suspected cases were tested. We fitted the random walk model described above to data from individuals in group *T*, and with this, then inferred the test results for the individuals of group *U*. We then evaluated the age distribution of cases obtained from the combined test (real positive test results from group *T* plus estimated positive test results from group *U*) to the real case distribution obtained from the test for group *N* using equivalence testing [19]. We use a two-one-sided test (TOST) approach, with the goal of assessing that the two distributions are equivalent (i.e. ‘similar enough’). This methodology assumes that the distributions are different, and thus rejecting the null hypothesis means that the two distributions are equivalent; it has been used in the past in pharmacokinetics to compare different treatments [20, 21]. However, it requires that we specify an equivalence criterion; here we define equivalence as the error in the estimation of cases across all age classes below 5% of the total number of cases, hereafter *D*1 equivalence as we will consider an alternative criterion later on (*D*2):
2
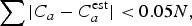
where *C*_*a*_ is the real number of cases at age *a* among all tested individuals *N* and 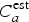 is the estimated number of cases at age *a*. The random sub-sampling was performed 100 times.

#### Minimum estimated number of serological tests

Cross-validation approaches such as the one described above provide an approach to estimating average error [22]. Thus, if we vary the proportion of individuals tested (*P*_tested_ = *T*/*N*), we can use the same cross-validation technique to estimate the minimum proportion of individuals that need to be tested to correctly infer the age distribution, i.e. reject the null hypothesis that the two distributions are different. In the previous section, we used *D*1 equivalence, error across all age bins below 5%, we now consider a more operational definition, hereafter *D*2: two age distributions were defined as equivalent if the cumulative number of cases up to the age bin where 80% of all cases are present have a discrepancy below 10%. First, we need to find the age bin where 80% of cases among tested individuals *N* are, *a*_*u*_, such that:
3
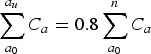
where *a*_0_ is the first age bin, *N* is the last age bin and *a*_*u*_ is the age bin up to which 80% of all cases are contained. We can then formulate a discrepancy below 10% in cumulative number of cases between our estimate and the data as:
4
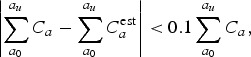
where *C*_*a*_ and 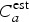 are as above, but the sums are only between the first age bin and *a*_*u*_. We chose this definition since from a programmatic point of view, identifying ages where most cases occur is important for choosing an appropriate intervention age range.

To iterate between different values of *P*_tested_, we adopted a simple bisection algorithm, which converges to the minimum number of tests needed relatively fast (more details are available in Supplementary material 1). For tractability, the IgM test is assumed to be perfect (100% sensitivity and specificity) in this section, as we are using the test results as the ‘gold standard’ to evaluate the cross-validation.

To asses the usefulness of our approach, we also estimated the minimum number of serological tests (*P*_tested_) needed for equivalence in a static confirmation rate model when a single age-independent confirmation rate is used to infer untested individuals, i.e. all untested individuals in all age bins have the same (the average) confirmation rate. We compared the minimum *P*_tested_ values to achieve equivalence in the age-specific model and the static confirmation rate model.

## Results

### Reconstructing inferred patterns

**Fig. 2.**
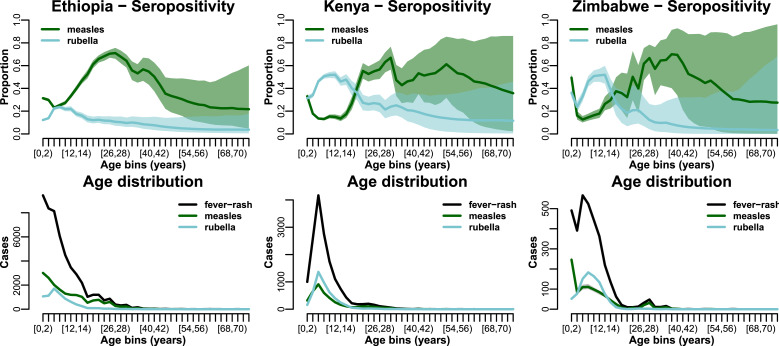
Seropositivity over age and age distribution of cases in the three countries studied. Top row shows the serological confirmation rate estimated from all tested individuals in each country for measles (green) and rubella (blue). Bottom row show the age distribution of all fever–rash cases (black) and the estimated (tested positive and estimated as positive) cases for measles (green) and rubella (blue).

In all three countries studied, the proportion of serological tests that confirmed measles infection in children decreases from the lowest age group (up to 2 years) to around age 15 (Fig. 2 top – green). This decrease is particularly strong in Zimbabwe, where the percentage of tested individuals that were confirmed as measles IgM-positive decreases from around 50% to 15%. In the two countries with higher measles vaccine coverage (Zimbabwe and Kenya), the percentage of measles laboratory confirmation for children below 15 years with fever–rash symptoms is well under 20% except in the first age class. The peak in serological confirmation is in individuals over 25 years of age (Fig. 2 top), while in older age classes, above 30–40 years, the predictions are uncertain because of low sample sizes. Notably, rubella serological confirmation shows a contrasting pattern of age incidence with measles, with the highest confirmation proportion in children between 5 and 15 years of age and dropping thereafter (Fig. 2 top – blue).

In all three countries, fever–rash cases occur mostly in the youngest ages (Fig. 2 bottom – green), due in part to most estimated measles and rubella cases being concentrated in children below 15 years. Indeed, this is not necessarily due to measles alone, as there are some disparities in the lower age groups between fever–rash cases and estimated measles cases – this is particularly apparent in Zimbabwe where many of the suspected measles cases, once corrected for by the age-specific confirmation rate, are re-classified as non-measles cases.

**Fig. 3.**
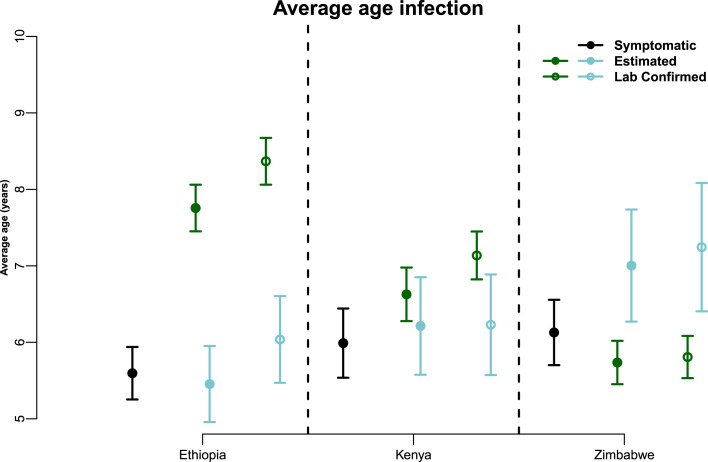
Mean age of infection in all three countries. Average age of infection for symptomatic (all fever–rash) cases in black; tested positive only are the hollow points in green for measles and blue for rubella; average age of infection for estimated cases are full points in green for measles and blue for rubella.

Mean age of suspected (symptomatic) cases does not change significantly over the three-country gradient of endemicity (Fig. 3). But the mean age of laboratory-confirmed, and estimated, cases changes in the directions that we would expect – mean age of measles cases increases as endemicity decreases (or coverage increases). Interestingly, mean age of rubella cases, both sero-confirmed and estimated, decreases as measles endemicity decreases, which could reflect on their relative contribution to the symptomatic cases. Syndromic cases are therefore not reflective of the gradient across countries because, within a country, suspected cases are less representative of measles as coverage increases.

**Fig. 4.**
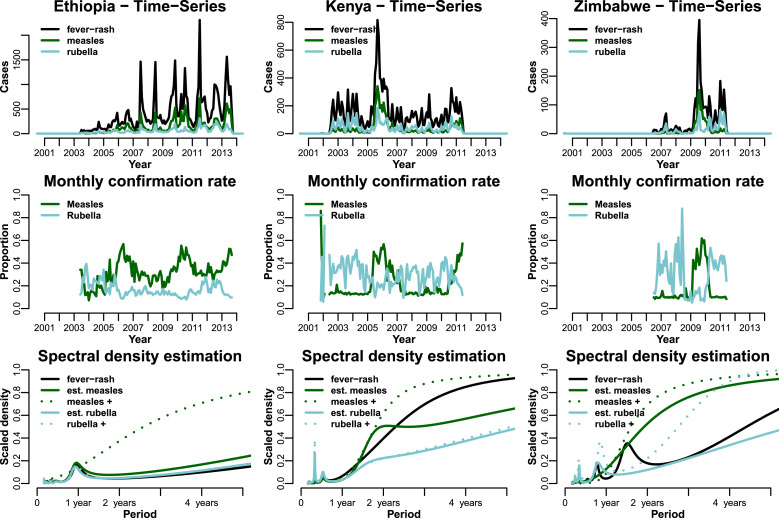
Time-series analysis for all three countries. Top row shows the time series for all fever–rash cases (black), estimated measles (green) and rubella (blue) cases. Middle row is the time series for the monthly serological confirmation for measles (green) and rubella (blue). Bottom row is the spectral density plots (*x*-axis is the period); we illustrate the symptomatic cases (black), tested positive as dotted lines (green for measles, blue for rubella) and solid green/blue lines for measles/rubella for the estimated number of cases.

Ethiopia exhibits recurrent, approximately yearly fever–rash (mainly due to measles) outbreaks (Fig. 4 – top left). However, when examining the frequencies, the measles- and rubella-confirmed cases fail to show the regular annual signature in Ethiopia (Fig. 4 – bottom left), where we know there really is a strong annual signature. This is not surprising given that laboratory sample collection is generally opportunistic and it is often biased to places that are higher performing and less likely to have a regular annual signature. On the other hand, the fever–rash and the age-corrected estimated time series do show the regular annual signature. This suggests that there is a benefit in using syndromic surveillance as laboratory-confirmed cases alone (in the absence of a formalised sampling strategy) would mask this pattern.

Kenya and Zimbabwe had a higher measles vaccination coverage, and only two and one outbreak, respectively, in the period is studied (Fig. 4 – top). There are no regular outbreaks, and the laboratory-confirmed cases tend to reflect the broad temporal patterns. Moreover, the time series in both these countries show large spikes in fever–rash cases; therefore, laboratory confirmation is important in evaluating whether spikes in syndromic cases are really due to measles or rubella. It is important to note that, at present, we respond to the former with vaccination, and not to the latter.

Monthly measles cases are estimated to account on average for only a small fraction of the total number of monthly fever–rash cases, from ~30% in Ethiopia to ~10% in Zimbabwe, except during the months with peak number of cases where this ratio can rise significantly in all three countries (Fig. 4 – middle row). The confirmation rate is not constant over time and can be fairly different in alternative settings, so assuming a fixed value of fever–rash cases as measles may lead to inappropriate inference. Moreover, the shift in confirmation rate is more drastic in lower endemicity countries.

### Cross-validation and minimum number of individuals tested

Assuming conservatively that 50% of the individuals were not tested, and allowing a 5% error in the estimation of true measles cases across all age bins (*D*1 equivalence), the TOST *P*-value is <10^−16^ in all three countries. This indicates that testing half of the individuals is enough to obtain ‘equivalent’ age distributions under our first definition of equivalence.

The minimum proportion of cases that need to be tested, *P*_tested_, to achieve equivalent age distributions is below 10% in all three countries. If we instead use a more programmatic definition of equivalence, focused on characterizing core ages of measles incidence, the 80th percentile (*D*2 equivalence), then the *P*_tested_ is 15% or less in all three countries (Table 2). When a similar approach is taken with an age-independent confirmation rate, the minimum number of individuals that need to be tested can be as high as 84% (Kenya). This suggests that the age-specific model needs around an order 10 less data to correctly characterise measles infection in the population.

**Table 2.** Summary of minimum number of individuals tested, *P*_tested_, for both definitions of equivalence, *D*1 and *D*2, in our age-dependent model and an age-independent model

Country	Age-specific model		Age-independent model	
	*D*1(%)	*D*2(%)	*D*1(%)	*D*2(%)
Ethiopia	3.9	6.1	51	35
Kenya	1.5	10	26	68
Zimbabwe	10	15	60	37

## Discussion

Here, we explored the power of limited age-specific serological confirmation to shed light on true patterns of incidence over age in syndromic fever–rash surveillance. In all three countries investigated, the proportion of suspected measles and rubella cases confirmed as IgM-positive is strongly age-dependent. While the general pattern of age-specific confirmation for measles and rubella is similar across countries – lowest confirmation rates for measles and highest for rubella between 3 and 15 years – the absolute rates are country-specific. Thus, inference about the age distribution of measles cases based solely on clinical cases could lead to significant biases. Specifically, our analysis shows that symptomatic surveillance alone can lead to spurious inference about the mean age of infection, with serologically confirmed cases and estimated number of cases showing the trends that we would expect. However, encouragingly, we find that relatively low levels of serological testing, below 15%, are needed to correctly estimate the age distribution of cases.

Across strikingly different patterns of measles age incidence, our analysis shows both the relative power of syndromic surveillance alone, but also how these differences can be leveraged to improve interpretation of surveillance results in settings where not every sample can be tested. The correction we propose can be used to both refine estimates on disease burden in a population; but also provides an insight into the returns on investments in surveillance effort, and particularly efforts put towards serological testing, for broadly differing epidemiological contexts.

As an example, to understand return on surveillance efforts in a particular setting, one valuable variable used is the number of measles cases found per fever–rash case. Our approach strengthens the inference around this variable. Across the different settings that we evaluated, we found that in endemic countries (Ethiopia), about one-third of the fever–rash cases were due to measles. By contrast, in countries with higher routine coverage, this ratio was much lower, due to the general absence of measles. However, in these measles non-endemic countries, the serological confirmation of measles increases dramatically during the periods with high incidence of fever–rash cases, which suggests a positive correlation (i.e. Fig. 4 – Kenya). Conversely, countries with high coverage of measles vaccine have higher rates of fever–rash cases caused by rubella, although in general we estimate around half of the monthly fever–rash cases as non-measles/rubella. This also highlights the fact that, even in countries with high measles vaccination coverage, an increase in fever–rash cases can be driven by an increase in measles or rubella incidence (Fig. 4 – Zimbabwe first and second peaks in fever–rash cases, respectively).

On the other hand, the high number of symptomatic cases in the younger age groups means that the absence of serological information can result in an underestimate of the mean age of infection (Fig. 3), exaggerating the total number of cases, while under-representing older age groups. This could lead to inadequate assessment of the current control strategies’ impact, as well as improper implementation of newer ones. Conversely, assessing disease burden purely with serology, particularly when not all cases can be tested, could potentially translate into missing the temporal signal, as we showed in the spectral density plot for Ethiopia (Fig. 4). The state-space models currently used to estimate global burden of measles mortality rely on the number of reported syndromic cases [23]; our analysis suggests that the resulting estimates of reporting rate are inherently confounded with the specificity of the case definition, which may vary among countries. Thus, one could first correct syndromic surveillance via the estimated age-specific confirmation rate prior to the use of state-space models (a hierarchical model framework could be used to account for uncertainty in the corrected time series of confirmed cases).

A ‘hybrid’ approach that combines syndromic surveillance and laboratory confirmation for a subset of cases may be possible for many pathogens in many different settings [24]. Using only laboratory-confirmed cases means that large amounts of data might be discarded (i.e. in our data, in Ethiopia, only ~55% of the cases where serologically tested); furthermore, for measles, outbreaks may not be adequately captured, because, following WHO guidelines [10], serological tests are not performed during large outbreaks. On the other hand, using only syndromic cases (here, epidemiologically linked fever–rash cases) is likely to result in confounding effects with other diseases. For example, in our analysis, rubella shapes much of the fever–rash incidence in Kenya and Zimbabwe. In general, syndromic surveillance for symptoms that can be caused by many agents (e.g. for diarrhoeal disease, fever–rash, etc.) may be of limited utility alone. To be of public health relevance, one must understand how this syndromic incidence relates to a particular aetiological agent – e.g. understanding the impact of rotavirus vaccination will require some insight into the link to diarrhoeal disease. The methods we suggest here provide one avenue to recovering core epidemiological patterns such as disease burden, or patterns of incidence over age, while minimizing the confounding effects of other diseases.

In relevant public health settings, testing every suspected case is likely to be logistically infeasible. Here, we focused on the testing required to achieve a minimum accuracy level; however, defining this minimum accuracy level is subjective. We proposed two different definitions of equivalent age distributions: estimating the burden in the population (with a 5% error) and estimating the burden in the core ages in which 80% of cases occur (with a 10% error), which we believe is a more useful definition from a programmatic point of view. Our cross-validation results indicate that testing only half of the syndromic cases yields strong inference into the total number of cases (TOST *P*-value <10^−16^ in all three countries). This arises first because the random walk framing allows estimates for age bins with small numbers of individuals and thus potentially high uncertainty to be informed by neighbouring bins; and second, because the age distribution is a very good predictor of seropositivity, given its role in infection risk. Our model also improves the estimation of age distribution when compared with a simpler approach with age-independent confirmation rate, which requires testing at least twice as many syndromic cases (Table 2).

One surprising result of our analysis is that even in countries where measles is not endemic, we find in high incidence measles months, a high number of non-measles fever–rash cases also registered. While this could be due in part to the uncertainty in the testing itself (i.e. false negatives) that are taken as non-measles, our estimates of ‘true’ cases formally accounts for the potential for false negatives; and furthermore, the sensitivity of the test itself is quite high. Both these lines of evidence suggest that other diseases are causing the symptoms seen in these data. Rubella is a clear candidate as an alternative cause for the fever–rash symptoms, since it has a similar route of transmission and affects children of a similar age. However, data from the rubella serological testing suggest that rubella is not the whole story, thus intriguingly suggesting the presence of another pathogen whose incidence is increased with measles incidence. Alternatively, it could be a sampling bias, where more syndromic cases are captured in the system when a disease outbreak is ongoing.

To conclude, we developed a simple model to leverage the age-specific confirmation proportion, here based on IgM serological tests, to infer true measles and rubella cases from suspected, syndromic cases. Our results have general relevance for an array of syndromic surveillance systems: we have shown that, taking advantage of the age-specific confirmation proportion, we can inform policy and surveillance measures by testing only a small proportion of individuals.

## Supplementary material

For supplementary material accompanying this paper visit http://doi.org/10.1017/S0950268818002054. click here to view supplementary material
