# Supplementary material for: Improving measles incidence inference using age-structured serological data
Source: Epidemiol Infect. 2018 Aug 6;146(13):1699–706. doi: 10.1017/S0950268818002054 (PMC9507956; doi:10.1017/S0950268818002054)
Supplement: Supplementary file 1 [file S0950268818002054sup001.pdf]

Supplementary material 1 - Details of model implementation

J. M. Prada *et al.*

June 25, 2018

In this supplementary, the methodology to estimate the age-specific confirmation rate is first described, followed by a section on the cross-validation and equivalence testing. Age-specific confirmation rate was estimated using an auto-regressive (AR) model, which required dividing the tested individuals into age groups (total n=38 of two-year age bins); all individuals above 74 years were collapsed into the latest age bin. Model cross-validation to find the minimum number of syndromic cases that need to be tested required matching two distribution, the equivalence of which was assessed using a two-one-sided test (TOST).

1 Autoregressive model

For each country, we run an autoregressive AR(1) process to estimate serological confirmation with age. This requires grouping the individuals into age groups and assumes that the confirmation proportion in one age group depends on the previous age group.

The probability that a syndromic fever-rash case is confirmed as measles by IgM testing for a given age group, $P_{age}^{measles}$, can be estimated as,

$$logit(P_{age+1}^{measles}) \sim Normal(logit(P_{age}^{measles}), \sigma_m) \tag{1}$$

The logit is used as the link function, and un-informative priors are employed for both σ_m and $P_1^{measles}$ (first age group). The analogous equation was used, independently, to estimate rubella serological confirmation with age.

The model was fit in R [1] using the Gibbs sampler package "jags" [2] and "runjags" [3]. Two independent chains were run, with 10000 samples and a burn in period of 1000. Convergence was verified using the Gelman and Rubin's convergence diagnostic [4].

2 Cross-validation

To validate our model outputs, we employed a repeated random sub-sampling validation design. We take all tested individuals, N, into consideration and assume a proportion of them are not tested, U, while we have test results for the remaining, T ($N = T + U$). We then run our auto-regressive model on the tested individuals T only, estimate the results for the ones assumed untested U, and compare how the age distribution of all cases in N compares to the truth. It is important to note here that we have to assume the test results of individuals N the truth ("gold standard").

If most individuals are tested, we expect both distributions to be fairly similar and conversely if very few individuals are tested, we would have a low power to properly estimate the age distribution. Assessing if the distributions are similar enough (i.e. equivalent) is discussed in more detail in the next section. To estimate the minimum proportion that needs to be tested to obtain good estimates (i.e. equivalence in the age distributions), we iterate through different number of individuals tested with a simple bisection method:

1. Choose the number of individuals assumed untested U and the number of individuals tested T.

2. Run the auto-regressive model on individuals T to estimate the age specific sero-confirmation rate. This step is run multiple times ($n = 100$), randomly choosing different individuals as T and U.

3. For each auto-regressive model above, we estimate test results for the assumed untested individuals U. This is done also multiple times ($n = 100$).

4. A median age distribution is collated from all the repeats and equivalence is tested using TOST (See below).

5. Check the p-value of the TOST. If below 0.05,

(a) Check for convergence, if the "step size" is small enough (see below), then the algorithm finishes (exits).

(b) Otherwise the number of individuals T is reduced.

If the p-value of the TOST is above 0.05, the number of individuals T is increased.

6. go back to step 2

A step is considered small enough if the absolute difference in individuals tested between this iteration and the previous $< 1\%$ of N. T is reduced or increased using a bisection method, which at each iteration reduces the step size (i.e. the amount the size - number of individuals in the T group - is increased or decreased is smaller after each iteration). At step 5, if the p-value is below 0.05, we assume the two distributions are equivalent, therefore we can decrease the number of individuals tested T. Conversely, if the p-value is above 0.05, we assume the two distributions are not equivalent, and therefore we need to increase the number of individuals tested T. The method is run until it converges (i.e. exits) or reaches a maximum of 50 iterations; this maximum was never reached in our simulations.

3 Equivalence testing

When trying to compare two distribution, the most commonly used statistical test, Kolmogorov-Smirnov (K-S), takes the null hypothesis as one distribution being drawn from the other one (the reference distribution). Therefore rejecting the null hypothesis mean they two distributions are different. However, failure to reject the null does not mean that the two distributions are the same/equivalent in the sense we need it for this work. See the figure S1 for two age distributions which are not equivalent in the sense we would like, but with a K-S test we fail to reject the null (note that for a K-S test the cumulative distributions were used).

We therefore use a two-one-sided test (TOST) approach, with the goal of assessing that the two distributions are equivalent (i.e. "similar enough"), [5]. This methodology assumes that the distributions are different, and thus rejecting the null hypothesis means that the two distributions are equivalent, it has been used in the past in pharmacokinetics to compare different treatments [6, 7]. It however requires specification of an "equivalence criterion" -

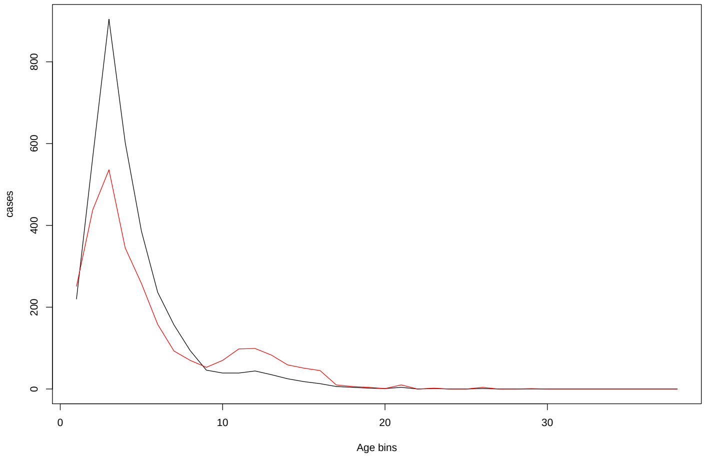

Figure S1: Two age distributions which we consider different, but with a Kolmogorov-Smirnov test we fail to reject the null. We therefore require an alternative test.

what is the condition for the two distributions to be considered equivalent. We defined two different measures of equivalence, which we called $D1$ and $D2$ equivalence.

The first definition of equivalence, $D1$, is taken as the error in the estimation of cases across all age classes below 5% of the total number of cases. The aim with this definition is making sure that all age bins have approximately the same number of cases. It can be calculated with the following equation,

$$\sum |C_a - C_a^{est}| < 0.05N \tag{2}$$

where C_a is the real number of cases at age a among all tested individuals N and C_a^{est} is the estimated number of cases at age a.

The second definition of equivalence we considered, $D2$, is taken as the cumulative number of cases up to the age bin where 80% of all cases are present have a discrepancy below 10%. The aim with this definition is to focus on the younger age classes, where most cases occur. We call this equivalence more programmatic, as measles and rubella vaccination programs target young individuals. Since the age bins are two years wide, and we only consider the first few age groups, a discrepancy of 5% would have been too restrictive, and thus we chose 10%.

First, we need to find the age bin where 80% of cases among tested individuals N are, a_u, such that,

$$\sum_{a_0}^{a_u} C_a = 0.8 \sum_{a_0}^{n} C_a \tag{3}$$

where a_0 is the first age bin, n is the last age bin and a_u is the age bin up to which 80% of all cases are contained. We can then formulate a discrepancy below 10% in cumulative number of cases between our estimate and the data as:

$$|\sum_{a_0}^{a_u} C_a - \sum_{a_0}^{a_u} C_a^{est}| < 0.1 \sum_{a_0}^{a_u} C_a \tag{4}$$

where C_a and C_a^{est} are as above, but the sums are only between the first age bin and a_u.

References

[1] *R: A Language and Environment for Statistical Computing*. R Foundation for Statistical Computing, 2008. R Development Core Team.

[2] M. Plummer, "JAGS: A program for analysis of Bayesian graphical models using Gibbs sampling," in *Proceedings of the 3rd International Workshop on Distributed Statistical Computing*, 2003.

[3] M. J. Denwood, "runjags: An R package providing interface utilities, model templates, parallel computing methods and additional distributions for MCMC models in JAGS," *Journal of Statistical Software*, vol. 71, no. 9, pp. 1–25, 2016.

[4] A. Gelman and D. B. Rubin, "Inference from iterative simulation using multiple sequences," *Statist. Sci.*, vol. 7, pp. 457–472, 11 1992.

[5] E. Walker and A. S. Nowacki, "Understanding Equivalence and Noninferiority Testing," *Journal of General Internal Medicine*, vol. 26, pp. 192–196, Feb. 2011.

[6] D. J. Schuirmann, "A comparison of the two one-sided tests procedure and the power approach for assessing the equivalence of average bioavailability," *Journal of Pharmacokinetics and Pharmacodynamics*, vol. 15, no. 6, pp. 657–680, 1987.

[7] J. L. Rogers, K. I. Howard, and J. T. Vessey, "Using significance tests to evaluate equivalence between two experimental groups.," *Psychological bulletin*, vol. 113, no. 3, p. 553, 1993.
